# The Burden of Multiple Basal Cell Carcinomas: A Population-wide Study

**DOI:** 10.2340/actadv.v104.40112

**Published:** 2024-05-27

**Authors:** Johan KAPPELIN, Ingela AHNLIDE, Åsa INGVAR, Kari NIELSEN

**Affiliations:** 1 Department of Clinical Sciences Lund, Dermatology, Lund University Skin Cancer Research Group, Lund University, Lund; 2 Dermatology Department, Landskrona Hospital, Landskrona; 3 Skåne University Hospital, Lund; 4 Department of Clinical Sciences Helsingborg, Dermatology, Lund University, Lund; 5 Helsingborg Hospital, Helsingborg, Sweden

**Keywords:** basal cell carcinoma, epidemiology, skin cancer

## Abstract

Basal cell carcinoma (BCC) is a common skin cancer type and affected individuals are known to be at risk of developing multiple consecutive tumours. Research into BCC multiplicity has, thus far, been challenging, due to a lack of national registration. This registry-based cohort study aimed to analyse the occurrence of multiple BCCs in Sweden, and risk factors for subsequent primary BCCs. Data regarding all histopathologically verified, primary BCC tumours in Sweden from 2004 to 2017 was extracted from the Swedish BCC Registry. Risk of developing a subsequent BCC in relation to person-related factors was estimated with Cox regression analysis. Cumulative risk of BCC development after 1 or 3 earlier BCCs was estimated. In total, 39.9% of individuals with a registered BCC had at least 2 registered tumours. The risk of developing a subsequent BCC increased significantly in males, older age, and with residence in southern Sweden. The cumulative 5-year risk of developing an additional BCC after first diagnosis was approximately 30% in males and 27% in females and increased after multiple previous BCCs. This study showed the cumulative risk of a subsequent BCC to increase with a history of multiple BCCs, indicating the need for clinical surveillance in these individuals.

SIGNIFICANCE

Basal cell carcinoma is a common skin cancer type and affected individuals are at risk of developing multiple tumours over time, rendering a substantial impact on healthcare services. The common lack of a national registration policy has hampered research on the prevalence of basal cell carcinoma and further knowledge is essential to aid in prevention and management. The Swedish Basal Cell Carcinoma Registry, consisting of all biopsied basal cell carcinoma tumours in Sweden, gives us a valid estimate of the occurrence of multiple basal cell carcinoma in Sweden and thus makes an important contribution to the existing research.

Basal cell carcinoma (BCC) is the most common skin cancer type in fair-skinned populations (1). Its high incidence, together with the increasing incidence of cutaneous squamous cell carcinoma, impacts healthcare costs hugely (2). Notably, European studies have shown that the incidence of BCC is expected to increase continually (3). However, the true incidence and burden of this common tumour is difficult to estimate due to a widespread lack of national registration (4). We have previously published incidence numbers from the Swedish national BCC Registry (5). This registry includes all histologically verified, primary BCC tumours in Sweden, providing us with a valid estimate of the incidence trends. Our earlier study presented a person-based adjusted incidence rate of BCC in Sweden in 2017 of 405/100,000 person-years (European standard population, 2013), increasing by 1.8% annually since 2004. In the same study, a tumour-based incidence of 548/100,000 person-years was found with an annual increase of 3.0%, indicating, as expected, a high prevalence of multiple BCC in the population. The rates were higher in the southern part of the country, indicating a latitude gradient on BCC incidence.

It is well known that high-risk individuals often develop multiple primary BCCs. However, the true incidence of multiple BCCs is challenging to appreciate, because national registries rarely include more than 1 tumour in each person (4, 6). Consequently, previous studies on multiple BCCs are primarily based on data from smaller cohorts rather than nationwide registries.

We aimed to study the occurrence and possible risk factors for multiple BCCs, using the Swedish BCC Registry.

## MATERIALS AND METHODS

We performed a registry-based, nationwide, cohort study, using the Swedish BCC Registry, held by the National Board of Health and Welfare and consisting of all histologically verified, primary BCC tumours in Sweden since 2004 (7). The registry is further described in our previous publication (5). Pseudonymized data regarding tumour and person characteristics on all registered tumours in the BCC Registry for the years 2004–2017 was extracted and linked to extracted data from the National Cause of Death Registry. To investigate the effect of latitude on risk of multiple BCC, each included individual was assigned residency to the medical region in which the diagnosing pathology department was located (Fig. S1).

### Statistical analysis

Descriptive statistics were presented in tables as crude rates and proportions, together with data regarding missing information. Univariate Cox regression analysis was performed to estimate the association between age, sex, region of residence, and risk of a consecutive primary BCC after the diagnosis of 1, 2, and 3 previous BCC diagnoses respectively. Participants were followed from the date of first, second or third BCC until diagnosis of next tumour, death, or end of the study period on 31 December 2017. Individuals with registered date of death occurring before date of first registered BCC diagnosis, due to misregistration in the BCC Registry, were excluded from the survival analysis. Hazard ratios (HR) are shown with a 95% confidence interval (CI), comparing risk of a subsequent BCC tumour in comparison with a reference category. A *p*-value < 0.05 indicated significance. No data were missing in the analysed variables. Cumulative risk of developing a subsequent BCC tumour is depicted with Kaplan–Meier graphs. All analyses were performed in R v. 2.2.2 (8) (R Foundation for Statistical Computing, Vienna, Austria).

The study was approved by the regional ethics committee, Lund University (#2017/378, #2017/993).

## RESULTS

From 2004 to 2017, 579,890 primary BCC tumours were registered in Sweden in 306,551 separate individuals (47.6% male and 52.4% female). In total, 122,438 individuals (39.9%) developed multiple BCC tumours. About half of these were diagnosed with 2 BCCs during the study period, and the rest with at least 3 BCCs (**Table I**).

**Table I:** Number of patients with single and multiple basal cell carcinoma (BCC) tumours diagnosed, divided by sex, and tumour characteristics in patients with single vs multiple BCC tumours

Number of BCC tumours	Male *n* (%)	Female *n* (%)	Individuals *n*
1	85,554 (46.5)	98,559 (53.5)	184,113
2	31,874 (47.5)	35,264 (52.5)	67,138
3	12,120 (49.2)	12,529 (50.8)	24,649
> 3	16,238 (53.0)	14,413 (47.0)	30,651
Total no. of persons (%)	145,786 (47.6)	160,765 (52.4)	306,551
Total no. of tumours (%)	288,469 (49.7)	291,421 (50.3)	579,890
	Single BCC tumour, *n* (%)	Multiple BCC tumours, *n* (%)	
Nodular BCC	69,484 (37.7)	119,724 (30.3)	
Superficial BCC	32,303 (17.5)	81,872 (20.7)	
Micronodular/Infiltrative BCC	54,277 (29.5)	122,601 (31.0)	
Morpheiform BCC	8,080 (4.4)	25,050 (6.3)	
Metatypical BCC	1,072 (0.6)	2,382 (0.6)	
Information lacking	18,896 (10.3)	44,145 (11.2)	
Head/neck	102,483 (55.7)	218,223 (55.1)	
Trunk	56,823 (30.9)	112,486 (28.4)	
Upper limb	6,912 (3.8)	15,598 (3.9)	
Lower limb	10,457 (5.7)	32,483 (8.2)	
Information lacking	7,430 (4.0)	16,958 (4.3)	
Total	184,112 (100.0)	395,774 (100.0)	

The most common BCC subtype was nodular BCC for individuals with single as well as for those with multiple tumours. Among individuals with multiple BCCs, the proportion with nodular subtype was lower compared with individuals with single BCC (30.3% vs 37.7%). Meanwhile, the proportion of superficial BCCs was slightly higher in patients with multiple BCCs (20.7% vs 17.5%) (Table I). Regardless of whether the patient was affected by single or multiple BCC, the head and neck region was the most common site with 55.7% and 55.1% of registered tumours respectively. The second most common localization was the trunk, with 30.9% of registered tumours among individuals with single BCC and 28.4% among individuals with multiple BCC (Table I).

There was a coherence regarding characteristics of consecutively diagnosed tumours in the same individual. Around 40% of individuals with a nodular, superficial, or micronodular/infiltrative subtype in the first diagnosed BCC had the same subtype in the second tumour. The corresponding proportion regarding morpheiform BCC was 29.2% (**Table II**). A similar pattern was seen among third diagnosed BCCs, although to a lesser extent. Regarding tumour site, the same correlation was found, where 79.0% of individuals diagnosed with their first primary BCC in the head/neck area developed their second tumour on the same site. The corresponding number for the trunk, upper limb, and lower limb was 59.4%, 31.6%, and 49.6% respectively (**Table III**).

**Table II:** Distribution of histological type of basal cell carcinoma (BCC) 2004–2017 among second and third BCC, based on histological type of first BCC

Tumour 1	Tumour 2						
	Nodular BCC %	Superficial BCC %	Micronodular/Infiltrative BCC %	Morpheiform BCC %	Metatypical BCC %	Information lacking %	Total
Nodular BCC	41.8	18.7	27.6	3.5	0.5	7.8	38,046
Superficial BCC	28.4	40.1	20.3	2.7	0.4	8.1	23,543
Micronodular/infiltrative BCC	26.6	13.3	43.8	7.6	0.7	8.0	38,592
Morpheiform BCC	15.9	8.6	36.0	29.2	0.8	9.5	7,431
Metatypical BCC	24.2	12.0	31.3	8.0	13.9	10.6	661
Information lacking	24.0	13.8	26.0	5.2	0.6	30.5	14,164
Tumour 1	Tumour 3						
Nodular BCC	38.2	20.4	28.6	4.4	0.4	8.1	4,065
Superficial BCC	29.8	34.2	23.6	3.7	0.4	8.2	2,937
Micronodular/infiltrative BCC	29.4	17.0	37.8	6.5	0.6	8.7	4,284
Morpheiform BCC	24.9	13.2	38.6	14.1	0.7	8.6	918
Metatypical BCC	25.2	19.6	29.0	7.5	2.8	15.9	107
Information lacking	24.3	17.4	24.7	5.2	0.6	27.9	1,630

Darker coloured fields indicate higher proportions.

**Table III:** Distribution of tumour localization of basal cell carcinoma (BCC) 2004–2017 among second BCC, based on tumour localization of first BCC

Tumour 1	Tumour 2					Total
	Head/neck %	Trunk %	Upper limb %	Lower limb %	Information lacking %	
Head/neck	79.0	12.3	1.8	3.0	4.0	67,713
Trunk	26.8	59.4	3.9	5.9	4.0	35,884
Upper limb	28.1	26.9	31.6	9.5	3.9	4,581
Lower limb	22.6	19.3	4.6	49.6	3.9	9,020
Information lacking	44.8	25.9	3.3	7.1	18.9	5,230

Darker coloured fields indicate higher proportions.

### Risk of a new primary tumour

Of the 306,551 individuals with diagnosed BCC, 14 individuals were excluded from the Cox regression analyses due to misregistration. Included persons with 1, 2, and 3 earlier BCCs were followed for a median time of 4.5, 3.7, and 2.9 years, respectively. Median time to occurrence of a new BCC was 1.4, 1.5, and 1.2 years, respectively. In individuals diagnosed with 3 earlier BCCs, a new primary BCC was diagnosed within 2.8 years in 75.0% of cases. Patient and tumour characteristics are further described in Table SI.

Women had a significantly decreased risk, compared with men, of developing subsequent tumours, regardless of the number of previous BCC diagnoses (HR 0.89, CI 95% 0.88–0.90 after 1 previous tumour; HR 0.87, CI 95% 0.85–0.89 after 2 tumours; and HR 0.90, CI 95% 0.87–0.92 after 3 tumours) (**Table IV**). The male proportion of cases developing a new BCC grew with every consecutive diagnosis, from 49.2% males among individuals with 1 previous diagnosis that developed a second tumour to 52.6% males among individuals with 3 previous diagnoses that developed a fourth tumour (Table SI). The risk of developing a new primary BCC was greatest in the age category 65–84 years, regardless of the number of earlier BCC tumours (Table IV). Pertaining to the region of residence, risk of a new BCC was around 8% greater in the south of Sweden compared with the reference category, Stockholm-Gotland, regardless of number of previous BCC diagnoses. A slight but significantly decreased risk of a new tumour was found in the remaining regions, but only among individuals with 2 earlier diagnosed tumours (Table IV).

**Table IV:** Hazard ratio for developing a new primary basal cell carcinoma (BCC), depending on number of earlier diagnosed tumours, estimated by univariate Cox regression analysis

Variable	One earlier BCC HR (95% CI)^a^	Two earlier BCCs HR (95% CI)^a^	Three earlier BCCs HR (95% CI)^a^
Age			
< 45 years	0.62 (0.60–0.64)	0.66 (0.62–0.70)	0.86 (0.78–0.94)
45–64 years	0.80 (0.79–0.81)	0.82 (0.80–0.84)	0.92 (0.89–0.95)
65–84 years	1.00 (Ref.)*	1.00 (Ref.)*	1.00 (Ref.)*
≥ 85 years	0.99 (0.97–1.01)	0.92 (0.89–0.95)	0.91 (0.87–0.95)
Sex			
Male	1.00 (Ref.)*	1.00 (Ref.)*	1.00 (Ref.)*
Female	0.89 (0.88–0.90)	0.87 (0.85–0.89)	0.90 (0.87–0.92)
Region of residence^b^			
North	1.03 (1.00–1.06)	0.87 (0.84–0.91)	0.99 (0.93–1.05)
Middle Sweden	0.99 (0.98–1.02)	0.94 (0.91–0.97)	0.98 (0.94–1.03)
Stockholm–Gotland	1.00 (Ref.)*	1.00 (Ref.)*	1.00 (Ref.)*
West	0.97 (0.95–0.99)	0.95 (0.92–0.98)	1.00 (0.96–1.05)
Southeast	1.03 (1.00–1.06)	0.94 (0.91–0.97)	1.00 (0.95–1.05)
South	1.08 (1.06–1.10)	1.08 (1.05–1.11)	1.07 (1.03–1.11)

a Hazard ratio for developing a new primary tumour after diagnosis of 1, 2, or 3 earlier BCC tumours, in comparison with the reference category. Numbers are presented with a 95% confidence interval.

b Region of residence, defined by the medical region in which the diagnosing pathology laboratory was located, ordered from north to south.

CI: confidence interval, HR: hazard ratio, Ref: reference category.

* Differences within the group were significant, with a *p*-value < 0.05.

**Fig. 1:**
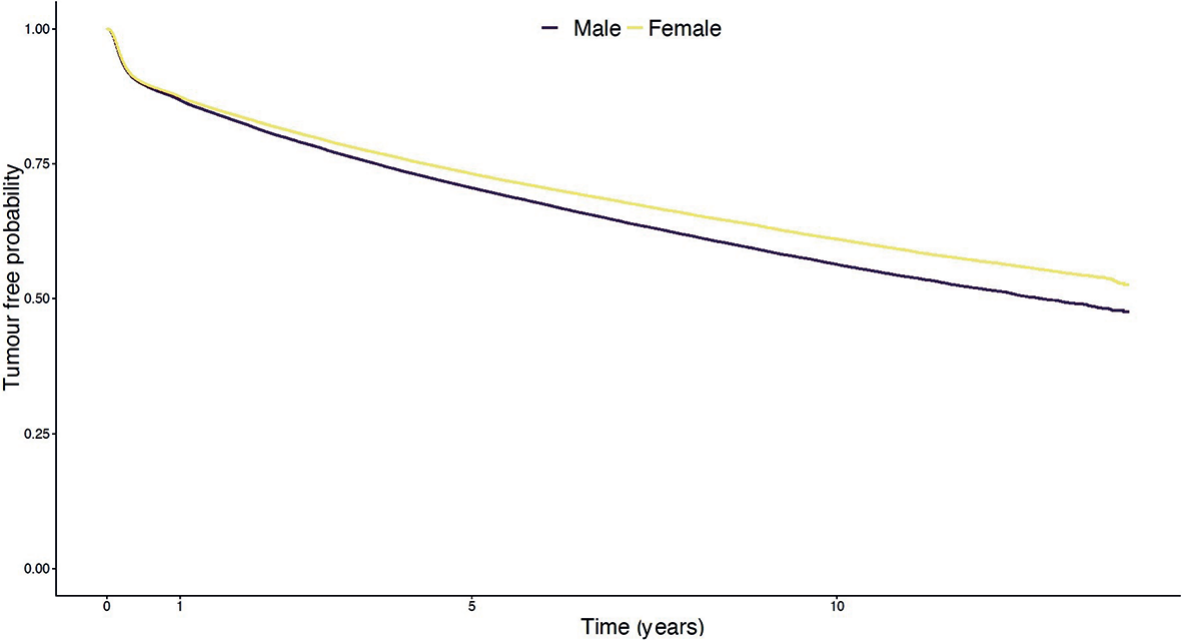
Cumulative risk of being diagnosed with a new primary basal cell carcinoma after being diagnosed with one earlier tumour, divided by sex. Presented in a Kaplan–Meier graph.

**Fig. 2:**
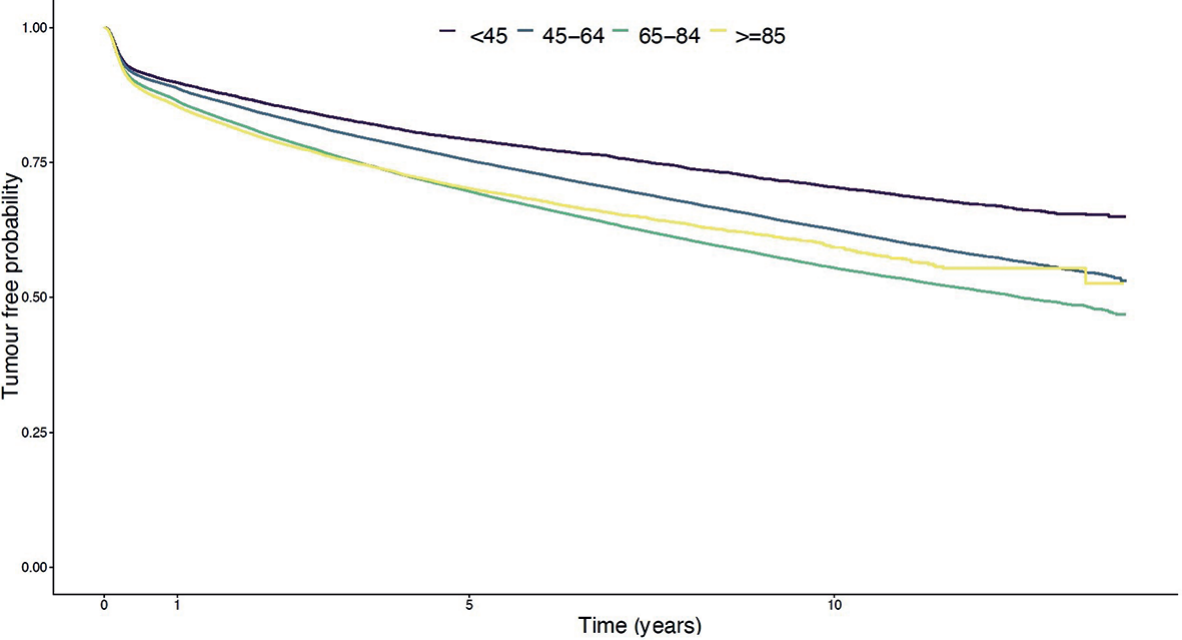
Cumulative risk of being diagnosed with a new primary basal cell carcinoma after being diagnosed with one earlier tumour, divided by age categories. Presented in a Kaplan–Meier graph.

Cumulative risk of developing a new tumour was approximately 27% in females and 30% in males 5 years after being diagnosed with the first BCC (**Fig. 1**). The cumulative 5-year risk of a new BCC increased with every additional BCC diagnosis. After 3 diagnosed BCCs, it reached approximately 43% in females and 48% in males (Fig. S2). The cumulative risk was lowest among the youngest and highest among individuals aged 65–84 years, as depicted in **Fig. 2**.

## DISCUSSION

In this study of BCC in the Swedish population during the period 2004 to 2017, multiple BCCs were registered in 41.3% and 38.7% of males and females, respectively. Risk of developing a new primary tumour was higher in males and increased with age and the number of earlier diagnosed BCC tumours.

In our study, 39.9% of registered individuals in the BCC registry were diagnosed with at least 2 BCC tumours. This relatively high number can be compared with a pooled proportion of 29.2% of individuals developing multiple BCC tumours, according to a meta-analysis by Flohil et al. from 2013 (9). After excluding studies using restrictive inclusion criteria in this meta-analysis, the proportion increased to 32.5%. Nevertheless, the tendency to develop new consecutive primary BCCs in Swedish individuals seems high, correlating to our findings of a high BCC incidence in Sweden, as shown in our previous study (5).

Individuals with a single BCC had a similar body distribution of tumours to individuals with multiple BCCs. Regarding tumour subtype, the fraction of superficial BCCs was somewhat higher in persons with multiple BCCs compared with persons with single BCC. Similar tendencies have been shown in several earlier studies (6, 10, 11). The lower proportion of nodular subtype among persons with multiple BCCs is slightly surprising. However, these numbers should be interpreted with caution, as registration of BCC in the Swedish BCC Registry is restricted to histopathologically verified tumours. Studies and clinical experience show that BCC tumours can be diagnosed and treated without histopathological verification (12, 13). This is reported primarily in low aggressive tumours such as nodular and superficial BCC, where adequate treatment can be achieved with non-surgical modalities (13). Furthermore, clinical experience suggests that non-surgical treatments are even more frequently used in individuals with multiple BCCs, rendering the proportion of clinically diagnosed tumours possibly even higher in this group. Thus, the proportion of particularly superficial BCC, and possibly also nodular BCC, is likely underestimated in this study, especially among patients with multiple tumours.

From clinical experience we have had the impression that patients with multiple BCCs repeatedly develop tumours of the same subtype and on the same sites. This clinical suspicion has been supported by scientific evidence in earlier studies (14), as well as in this study. Interestingly, this was also shown for aggressive BCC types in our study. Hence, clinicians should be vigilant regarding the higher occurrence of new aggressive BCCs in patients previously diagnosed with these subtypes. The biological basis for this phenomenon is unknown. Possibly, there are genetic predispositions in affected individuals that induce the development of specific tumour subtypes. Another possibility could be that predisposing external factors, such as sun exposure, differ between individuals and that a specific set of factors increase the risk of a particular subtype or a specific tumour localization, as speculated in earlier work (15). Indeed, the same pattern has been suggested in the development of BCC in relation to SCC (squamous cell carcinoma), where individuals with an earlier BCC diagnosis to a greater extent develop new BCC tumours, while individuals with an earlier SCC diagnosis primarily develop new SCC tumours (16). To further explain this phenomenon, in-depth studies on tumour biology and genetics are needed. Meanwhile, it is important to recognize that a history of BCC also increases the risk of other skin cancer types, such as malignant melanoma (17).

Furthermore, our study investigated the risk of developing a new tumour in relation to patient age, sex, region of residence, and number of earlier diagnosed tumours. The cumulative risk of developing a new tumour was estimated to be approximately 27% among females and 30% among males 5 years after first diagnosis of BCC. In the meta-analysis by Flohil et al., the mean 5-year cumulative risk of a new BCC tumour was presented as 36.2%, with risk estimates ranging from 11–50% (9). Thus, our population’s cumulative risk of developing new tumours was comparable to earlier findings. Meanwhile, the cumulative risk was approximately 43% among females and 48% among males for individuals with 3 earlier BCC diagnoses, indicating an increased risk after multiple previous tumours. Not many studies are available presenting this correlation. However, in a study by van Iersel et al. (18), the 5-year risk of developing a new BCC tumour was 28% in patients with only 1 previous BCC diagnosis and 48% among individuals diagnosed with multiple synchronous BCC tumours at the initial time of diagnosis. The finding of an increased risk of further BCC tumours in patients with a history of multiple BCC highlights the need for regular skin checks in these individuals. However, while most diagnoses of new primary tumours in our population are made within the first 3 years after the first diagnosis, the need for surveillance seems to be the most important during this interval.

The risk of developing a consecutive tumour increased with age, a finding presented in earlier studies as well (6, 19). The risk was slightly higher in males, and the male proportion of individuals with a new tumour grew for every consecutive tumour diagnosed. Similar findings have been presented in previous studies. The reason for the gender differences is not known but correlates to our earlier findings of a higher BCC incidence among males (5) as well as findings in previous studies, indicating an increased overall skin cancer risk among males (20). Possible explanations might include disparities in lifestyle factors or healthcare-seeking behaviour. However, biological differences between the sexes cannot be ruled out and further studies are needed to elucidate the basis of these findings.

Earlier studies, including our study of the Swedish BCC Registry (5), have shown BCC incidence to be related to latitude, with a higher incidence closer to the equator (19, 21). The present study could show a higher risk of developing a new tumour if living in the very south of Sweden, indicating that latitude gradient might also affect the risk of multiple BCCs. However, the remaining regions did not show the same clear trend. Notably, a significantly decreased risk in northern Sweden was seen only among cases with 2 earlier BCC diagnoses. While different hospitals could have a varying approach to BCC treatment with regard to non-surgical therapies, the proportion of BCC tumours reported to the BCC Registry might differ between various geographical areas. This might affect risk estimates in different parts of Sweden, making the geographical impact on the risk of multiple BCCs difficult to fully elucidate in the present study.

### Strengths and limitations

Our study is based on a comprehensive national registry of all primary, histologically verified BCC tumours from 2004 to 2017, in Sweden. The Swedish National BCC Registry is one of few registries incorporating consecutive BCCs in each patient. It is, therefore, an essential source of information regarding the presence of multiple BCCs in a predominantly fair-skinned population. Furthermore, this study presents estimates of cumulative risk of developing new primary BCCs, as well as correlations between patient factors and the number of earlier diagnosed tumours on the risk of a new BCC diagnosis.

The primary weakness of the present study is related to the structure of the Swedish BCC Registry, while the included cases are restricted to histopathologically verified tumours. As mentioned earlier, an unknown number of diagnosed BCC tumours are never biopsied and, therefore, are not included in this registry. Furthermore, the ratio of clinically diagnosed and not histopathologically verified tumours is probably higher among low aggressive tumours, such as superficial BCC or nodular BCC. Thus, it is likely that the number of diagnosed tumours in these categories is even higher than reported in the Swedish BCC Registry. Individuals were followed from the start of the Swedish BCC Registry and no lag-time was adapted. This might, theoretically, have affected our results.

### Conclusion

In this study of the nationwide Swedish BCC Registry, we present a high occurrence of multiple BCC tumours. The risk of developing new tumours was significantly affected by older age at first diagnosis and by male sex. Furthermore, data suggest the cumulative risk of a new primary tumour increases after multiple previous diagnoses. Thus, clinical surveillance in the first 3 years can be considered in patients with a history of multiple BCC.

## Supplementary Material

The Burden of Multiple Basal Cell Carcinomas: A Population-wide Study

The Burden of Multiple Basal Cell Carcinomas: A Population-wide Study
