# Supplementary material for: The Burden of Multiple Basal Cell Carcinomas: A Population-wide Study
Source: Acta Derm Venereol. 2024 May 27;104:40112. doi: 10.2340/actadv.v104.40112 (PMC11151493; doi:10.2340/actadv.v104.40112)

Supplementary material has been published as submitted. It has not been copyedited, or typeset by Acta Dermato-Venereolgica

Fig. S1. The six medical regions of Sweden. Each individual was assigned to the medical region in which the diagnosing pathology department was located.

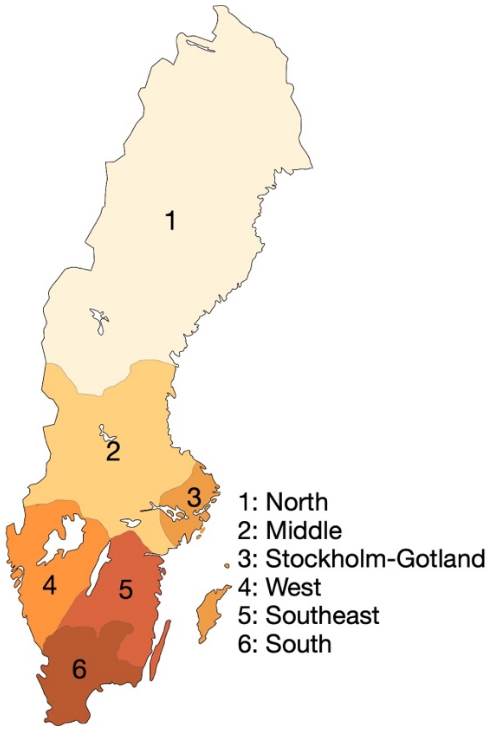

Fig. S2. Cumulative risk of being diagnosed with a new primary basal cell carcinoma after being diagnosed with three earlier tumours, divided by sex. Presented in a Kaplan-Meier graph.

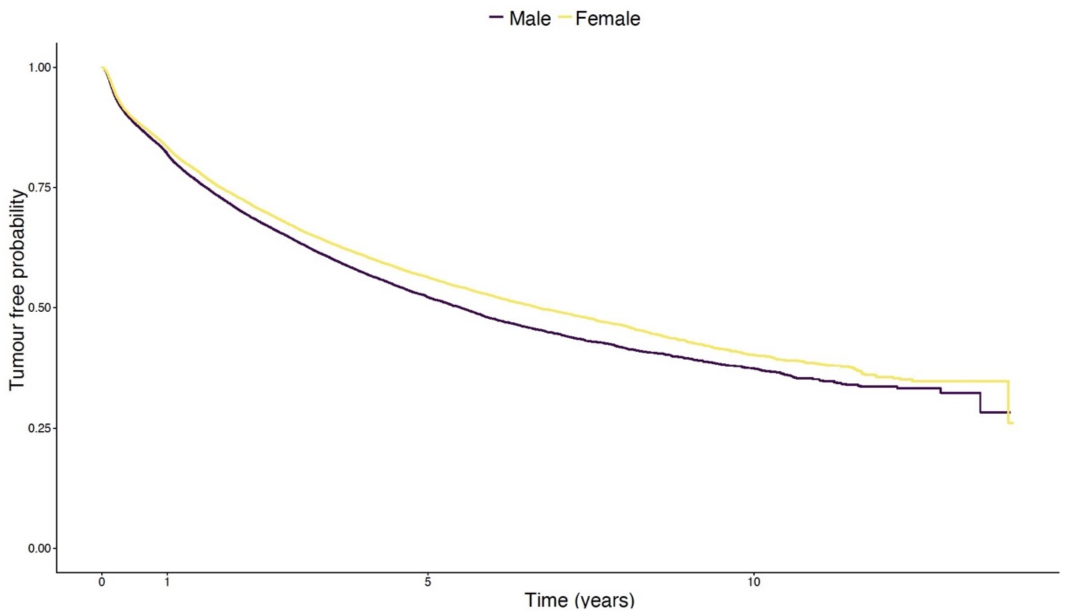

Supplement: The Burden of Multiple Basal Cell Carcinomas: A Population-wide Study [file ActaDV-104-40112-s1.pdf]
