# Supplementary material for: The Burden of Multiple Basal Cell Carcinomas: A Population-wide Study
Source: Acta Derm Venereol. 2024 May 27;104:40112. doi: 10.2340/actadv.v104.40112 (PMC11151493; doi:10.2340/actadv.v104.40112)
Supplement: The Burden of Multiple Basal Cell Carcinomas: A Population-wide Study [file ActaDV-104-40112-s2.pdf]

Supplementary material has been published as submitted. It has not been copyedited, or typeset by Acta Dermato-Venereolgica

Table SI. Baseline and follow-up characteristics divided by number of earlier diagnosed basal cell carcinoma (BCC) tumours

	**One earlier BCC**		**Two earlier BCC**		**Three earlier BCC**	
	No new BCC	New BCC	No new BCC	New BCC	No new BCC	New BCC
Follow–up time, years (median [IQR])[a]	4.5 [2.0, 7.9]	1.4 [0.3, 4.0]	3.7 [1.6, 6.6]	1.5 [0.4, 3.5]	2.9 [1.2, 5.3]	1.2 [0.4, 2.8]
Age at diagnosis (median [IQR])	71 [61, 80]	71 [62, 79]	74 [65, 82]	73 [65, 80]	76 [67, 83]	74 [66, 81]
Age categorized, n (%)						
<45	11 829 (5.4)	3 651 (4.1)	2 794 (3.4)	1 161 (2.9)	649 (1.9)	451 (2.2)
45–64	61 244 (28.0)	24 732 (28.1)	18 503 (22.5)	9 385 (23.4)	6 089 (17.7)	4 188 (20.0)
65–84	116 471 (53.3)	50 770 (57.7)	47 501 (57.7)	24 884 (62.1)	20 986 (61.1)	13 493 (64.4)
≥85	28 928 (13.2)	8 912 (10.1)	13 576 (16.5)	4 632 (11.6)	6 613 (19.3)	2 828 (13.5)
Sex, n (%)						
Male	102 414 (46.9)	43 366 (49.2)	39 861 (48.4)	20 370 (50.8)	17 341 (50.5)	11 016 (52.6)
Female	116 060 (53.1)	44 699 (50.8)	42 514 (51.6)	19 692 (49.2)	16 997 (49.5)	9 944 (47.4)
Region of residence[b] (%)						
North	15 706 (7.2)	6 220 (7.1)	6 397 (7.8)	2 678 (6.7)	2 423 (7.1)	1 406 (6.7)
Middle Sweden	40 395 (18.5)	15 623 (17.7)	14 914 (18.1)	6 618 (16.5)	5 829 (17.0)	3 253 (15.5)
Stockholm-Gotland	48 848 (22.4)	20 038 (22.8)	18 123 (22.0)	9 320 (23.3)	7 824 (22.8)	4 973 (23.7)
West	36 763 (16.8)	13 680 (15.5)	13 165 (16.0)	5 978 (14.9)	5 280 (15.4)	3 050 (14.6)
Southeast	22 544 (10.3)	9 209 (10.5)	8 894 (10.8)	4 190 (10.5)	3 618 (10.5)	2 278 (10.9)
South	54 218 (24.8)	23 295 (26.5)	20 882 (25.3)	11 278 (28.2)	9 364 (27.3)	6 000 (28.6)
Total, n	218 474	88 065	82 375	40 062	34 338	20 960

[a] Median time of follow-up among individuals with and without diagnosis of new primary tumour depending on number of earlier BCC diagnoses, depicted together with interquartile range (IQR). [b] Region of residence, defined by the medical region in which the diagnosing pathology laboratory was located, ordered from north to south.
